# Optical Coherence Tomography Angiography–Navigated Laser Photocoagulation of Retinal Hemangioblastomas in Patients With von Hippel–Lindau Disease

**DOI:** 10.1167/tvst.13.7.8

**Published:** 2024-07-09

**Authors:** Yannik Laich, Navid Farassat, Viviane Grewing, Daniel Boehringer, Felicitas Bucher, Peter M. Maloca, Thomas Reinhard, Stefan J. Lang, Hansjuergen Agostini, Michael Reich

**Affiliations:** 1 Eye Center, Medical Center – Faculty of Medicine, University of Freiburg, Freiburg, Germany; 2 Institute of Molecular and Clinical Ophthalmology Basel, Basel, Switzerland; 3 Moorfields Eye Hospital NHS Foundation Trust, London, United Kingdom; 4 Department of Ophthalmology, University Hospital Brandenburg, Brandenburg Medical School, Brandenburg an der Havel, Germany; 5 Augenärzte am Städel, Medical Practice for Ophthalmology, Frankfurt, Germany

**Keywords:** retinal hemangioblastoma, optical coherence tomography angiography, laser photocoagulation, von hippel–lindau, navilas

## Abstract

**Purpose:**

To describe optical coherence tomography angiography (OCTA)-guided navigated laser photocoagulation (LP) using the Navilas Laser System for treating retinal hemangioblastomas (RHs) associated with von Hippel–Lindau disease (VHLD).

**Methods:**

Patients with VHLD were screened using ophthalmoscopy and widefield OCTA. Detected RHs were classified with regard to tumor morphology (endophytic, sessile, exophytic, recurrent) and size. Then, 6 × 6- or 3 × 3-mm^2^ en face OCTA scans of the RHs were uploaded to the Navilas system, generating a merged image combining the scan and Navilas fundus photography. LP was planned by placing laser spots in the OCTA scan and executed with the Navilas system. Treatment efficacy was assessed by conducting OCTA scans immediately after LP and at follow-up visits.

**Results:**

Fifteen RHs were detected in 10 patients (median, one RH; range, one to four). Twelve RHs were treatment naive (exophytic [3], sessile [3], and endophytic [6]), and there were three recurrent RHs in pretreated areas. Total applied energy per tumor correlated with tumor size (*P* < 0.001). After a mean first follow-up of 3.6 ± 1.5 months (range, 0.9–5.3), nine RHs exhibited complete regression (60%), five partial regression (33.3%), and one no regression (6.7%). No correlation between tumor morphology and treatment success was observed (*P* = 0.32). However, a correlation between treatment success and tumor size trended toward significance (*P* = 0.08), with a 100% success rate observed for small RHs.

**Conclusions:**

OCTA-guided LP via the Navilas Laser System is a promising technique, especially beneficial for targeting small RHs. Combining OCTA and ophthalmoscopy improves tumor detection, underscoring the utility of this approach.

**Translational Relevance:**

OCTA-guided LP enables highly precise and safe treatment of early-stage RHs, minimizing possible complications caused by LP or the tumor itself.

## Introduction

Retinal hemangioblastomas (RHs) are common in over 70% of patients with von Hippel–Lindau disease (VHLD).^1^^–^^5^ Clinically, RHs present as reddish, globular, vascular lesions in the peripheral retina, less frequently in the juxtapapillary area.^6^^,^^7^ Three distinct growth types of RHs have been described, varying in their clinical appearance: endophytic, sessile, and exophytic.^8^^,^^9^

Although predominantly benign, RHs growth can induce pressure on neighboring structures, leading to severe complications such as massive exudation, hemorrhage, retinal detachment, loss of vision, or even loss of the eye.^7^ Therefore, RHs remain a major cause of visual impairment in patients with VHLD.^10^ Consequently, regular retinal screening of patients with VHLD is crucial to detect RHs at an early stage.

Ophthalmoscopy alone has a detection rate of 39%, which can be improved up to 89% applying further imaging modalities such as fluorescein angiography (FA).^11^^,^^12^ However, due to the need for dye injection, FA is an invasive, lengthy procedure with potential adverse effects,^13^ and it cannot be repeated on the same day to evaluate laser treatment success.

**Figure 1.**
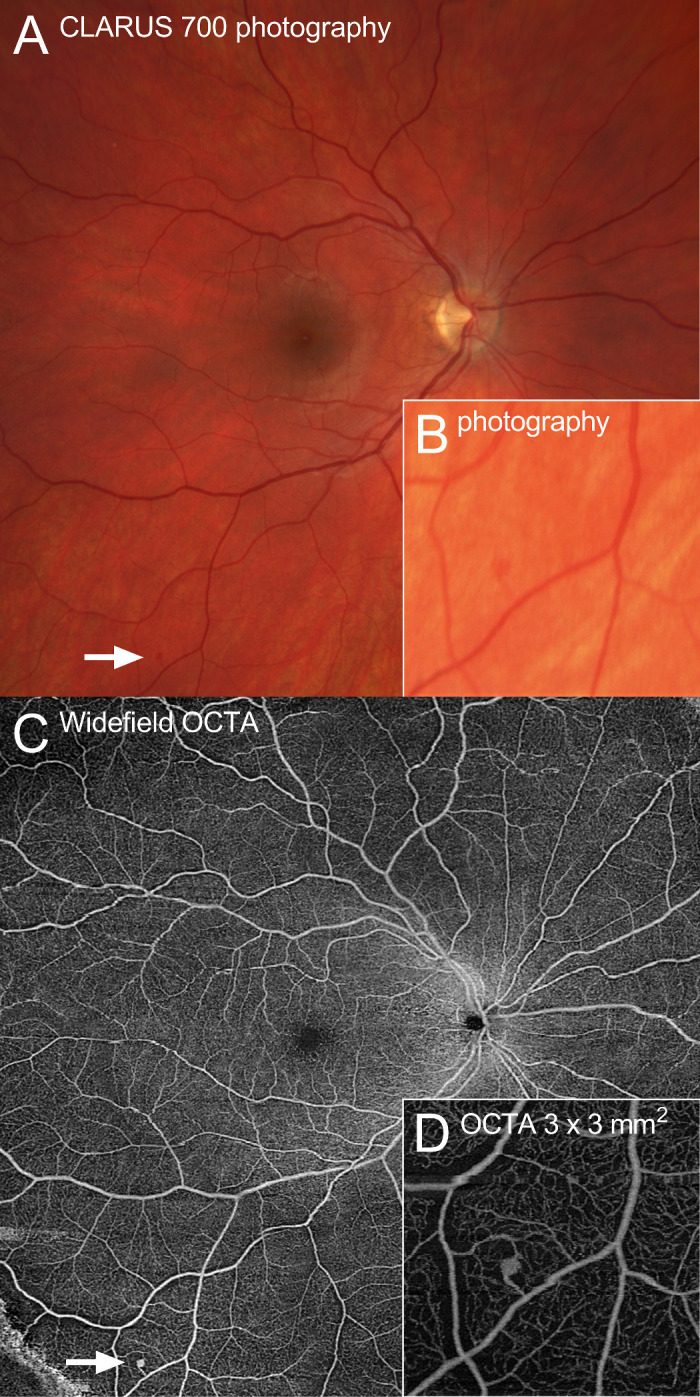
Multimodal imaging of a retinal hemangioblastoma. (**A**) A small *red dot* is barely recognizable on the CLARUS 700 fundus photography (*arrow*). (**B**) The small RH can only be recognized when the image is modified and enlarged. (**C**) In contrast, the RH lesion can be easily visualized as a bright *white dot* in the ZEISS PLEX Elite widefield OCTA (*arrow*). (**D**) In a high-resolution (3 × 3-mm^2^) scan, the entire RH lesion is recognizable as a tangle of vessels, even with afferent and efferent vessels, which are impossible to detect in the unmodified color fundus photography (**A**).

Optical coherence tomography angiography (OCTA) has been proposed for early RH detection, monitoring, and treatment evaluation.^14^^,^^15^ As a noninvasive, safe, and rapid imaging modality, OCTA allows for repeated imaging within a single session. Furthermore, OCTA shows higher sensitivity in detecting RHs compared to ophthalmoscopy alone, achieving a detection rate up to 92% in the retinal areas that can be imaged by OCTA.^11^ Consequently, OCTA is considered a valuable additional imaging modality, improving screening of VHLD patients. Moreover, OCTA can detect RHs that are barely visible in ophthalmoscopy (Fig. 1).

Early detection facilitates the implementation of focal laser photocoagulation as an efficacious and safe intervention for peripheral RHs,^16^ thereby averting disease progression and associated sequelae.^7^ Further treatment options include off-label use of belzutifan,^17^ cryotherapy, brachytherapy, transpupillary thermotherapy, proton irradiation, and intravitreal injection of vascular endothelial growth factor (VEGF) inhibitors.^18^^–^^20^

Beside conventional laser techniques, more modern laser procedures can be used today for treatment of RHs. The Navilas Laser System (OD-OS, Teltow, Germany), combining real-time fundus imaging and multimodal scan overlays, offers precise, efficient, and safe treatment planning and execution with automatic eye tracking. This system demonstrates a 92% hit rate on diabetic macular edema microaneurysms compared to 72% with conventional laser techniques.^21^ Furthermore, navigated laser photocoagulation has demonstrated similar efficacy and safety compared with conventional methods for treating retinal arteriolar macro-aneurysms, but it requires significantly lower total laser energy and results in fewer laser burns.^22^

Effective laser photocoagulation, using both conventional and Navilas systems, relies on precise localization of RHs. The feasibility of laser treatment for such small RHs identified through OCTA but minimally visible in ophthalmoscopy raises questions. Our study addresses this challenge by introducing a novel approach utilizing OCTA-guided navigated laser photocoagulation for RHs using the minimally invasive Navilas system.

## Materials and Methods

### Study Design

This was a prospective, single center study approved by the Institutional Ethics Committee (ID number 360/19), and it adhered to the tenets of the Declaration of Helsinki. Signed informed consent was obtained from all participants prior to inclusion.

### Study Population

The study cohort was comprised of individuals diagnosed with VHLD. Diagnosis was determined through genetic and/or clinical evaluations conducted at the VHLD clinic at the Center for Rare Diseases, University Medical Center Freiburg, Freiburg, Germany. Patient characteristics, including sex, date of birth, and *VHL* gene mutation were documented. Ten patients with VHLD exhibiting one or more RHs were included in this study.

### Inclusion and Exclusion Criteria

Only patients with a confirmed *VHL* gene mutation and/or meeting the clinical diagnostic criteria for VHLD were included. Patients with systemic diseases, such as diabetes mellitus or cardiovascular diseases, were excluded based on medical history. Furthermore, patients with concurrent retinal diseases or eyes with silicon oil tamponade were excluded. To ensure sufficient OCTA image quality, eyes with media opacities, such as advanced cataract, as well as eyes with a refractive error exceeding −7 diopters (D) were excluded. Only OCTA images with signal strength ≥7 out of 10 were included. The study did not include RHs that could not be imaged via OCTA due to a peripheral location or juxtapapillary RHs.

### Ophthalmological Examination

All patients attended their regular VHLD screening examinations including the following assessments. Automated refraction and best-corrected visual acuity were assessed. Pupils were dilated with 5-mg/mL tropicamide (Mydriaticum Stulln UD, Pharma Stulln GmbH, Stulln, Germany). Ultra-widefield photography was obtained using either the Optos P200DTx/A10650 (Optos, Dunfermline, UK) or CLARUS 700 (Carl Zeiss Meditec, Jena, Germany). Widefield OCTA scans were performed with the ZEISS PLEX Elite 9000 (Carl Zeiss Meditec). Five 12 × 12-mm^2^ retinal scans were assembled to create a widefield OCTA scan using the manufacturer's software. In cases of suspicious areas in the widefield OCTA scan, additional 3 × 3- and/or 6 × 6-mm^2^ scans were performed. Images were assessed by two independent investigators experienced with VHLD and OCTA (YL and MR). Subsequently, slit-lamp examination of the anterior segment and ophthalmoscopy were performed by two experts familiar with VHLD (YL, VG, FB, SJL, HA, or MR) using three different Volk lenses (SuperField, Digital Wide Field, or 90D, 20D, or 2.2 3-Mirror; Volk, Mentor, OH).

### Morphology of Retinal Hemangioblastomas

Classification of tumor morphology into exophytic, sessile, and endophytic growth patterns was based on their clinical appearance and distribution of flow signal in the OCTA B-scans. In cases of tumor recurrence within a previously treated retinal area, morphology classification was not performed.

### Tumor Size Measurement

The tumor size was estimated via ophthalmoscopy in relation to the optic disc size as follows: <0.25 disc diameter (DD), 0.25 to 0.5 DD, and >0.5 DD.

### Planning and Application of Laser Photocoagulation

**Figure 2.**
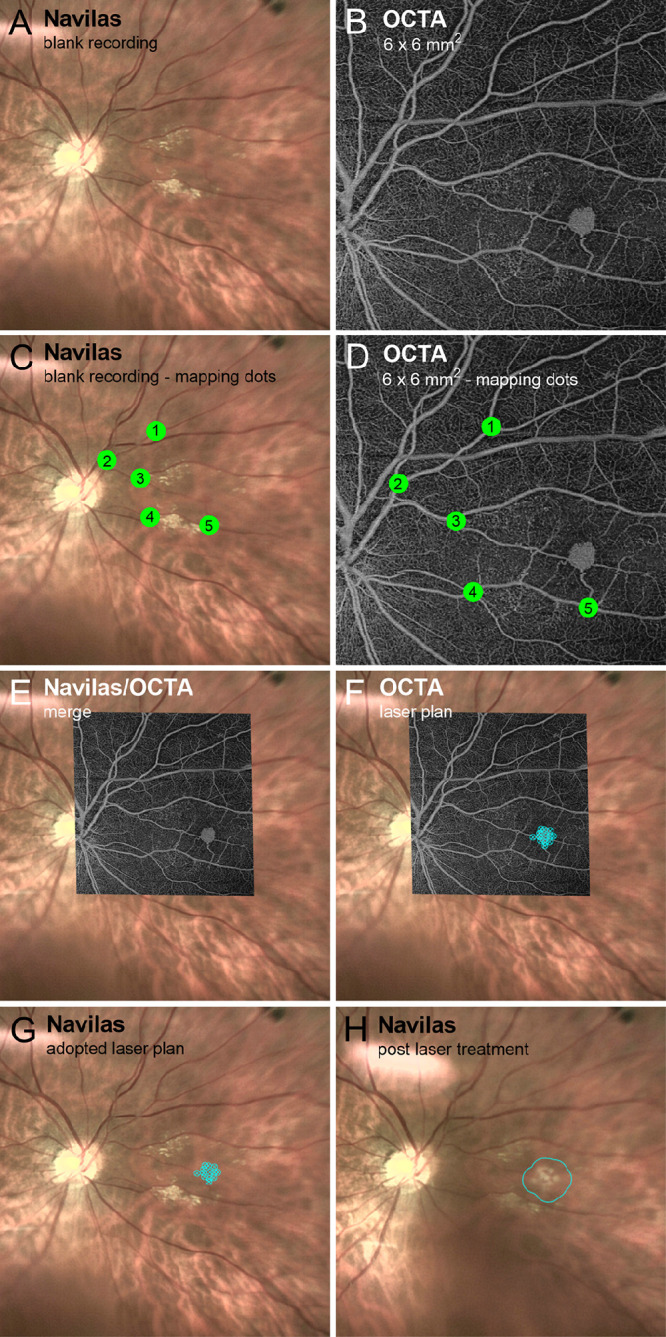
OCTA-enhanced Navilas laser treatment of retinal hemangioblastoma. (**A**) Ordinary Navilas fundus photography is shown. *Yellowish* hard exudates and a faint *reddish* spot corresponding to a RH that is barely recognizable can be seen. (**B**) In the corresponding OCTA, the RH is more clearly visible, whereas the hard exudates are less recognizable. (**C**) Planning and conducting the laser treatment for an exemplary RH included the manual marking of landmark vessels in the Navilas system (represented as consecutive numbers by two graders). (**D**) The same vessels were marked in the OCTA in an analogous manner. (**E**) In the next step, both images were superposed and scaled with the help of the manufacturer's software. (**F**) This made it possible to set the laser spot position precisely on the marked lesion (*blue area*). (**G**) The Navilas Laser System in treatment mode. (**H**) In Navilas photography, the postoperative result is visible as edema formation in the area of the original lesion (*blue-circled area*).

Planning and execution of OCTA-guided navigated laser photocoagulation using the Navilas for the treatment of RH is illustrated in Figure 2. Fundus photography of the affected eye was performed using the commercially available Navilas Laser System according to the manufacturer's protocol (Fig. 2A). Image acquisition and laser treatment were performed using a wide-angle lens in combination with an Ocular Mainster PRP 165 laser lens (Ocular Instruments, Bellevue, WA) placed on the corneal surface after local anesthesia with 4-mg/mL oxybuprocaine hydrochloride (OmniVision GmbH, Puchheim, Germany) and applying methocel 2% (OmniVision). After capturing the fundus images, OCTA scans (Fig. 2B) were uploaded to the Navilas software, and a merged image was created by manually labeling corresponding vascular junctions in both the photography and OCTA scan (Figs. 2C, 2D). Image import and overlay are commercially available software modules that can be added to the Navilas system without further specific adjustments. Two physicians (YL, MR) confirmed the accuracy of the overlay by visual inspection (Fig. 2E). Laser treatment was planned by placing multiple laser spots of 215-µm diameter on the RH and feeding vessels in the OCTA scan (Fig. 2F). This treatment plan was transferred to the fundus photography (Fig. 2G), and treatment was applied of 30-ms duration and individually adjusted power to ensure the appropriate treatment effect (Fig. 2H).

### Follow-Up

Directly after the laser treatment, another 3 × 3-mm^2^ and/or 6 × 6-mm^2^ OCTA scan of the treated area was performed. If no more OCTA flow signal was detected in the RH, the laser treatment was considered successful; a persistent flow signal indicated insufficient laser treatment. In case of insufficient laser effect, another laser photocoagulation was immediately performed with increased power. In the event of complications, such as bleeding, no further laser treatment was performed on the same day. First follow-up examinations were scheduled between 1 and 6 months after laser treatment, depending on the course of the laser session. The need and frequency of subsequent follow-ups depended on the course of the disease.

### Statistical Analysis

SPSS Statistics 20.0 (IBM, Chicago, IL) was used for statistical analysis. Mean, standard deviation, median, minimal and maximal values (range) were calculated for descriptive data analysis. Spearman’s correlation test (Spearman’s *r*) was used to analyze the correlation between tumor size and total laser energy used. The χ^2^ test was used to analyze the correlation between tumor morphology as well as tumor size and the outcome of the laser therapy. *P* < 0.05 was considered statistically significant.

## Results

### Patient Characteristics

A total of 18 eyes from 10 patients (seven females and three males) were included in this study. One patient presented with monocular status due to previous enucleation surgery. One eye from another patient was excluded due to a longstanding retinal detachment. Mean age at study inclusion was 41.5 ± 12.8 years (range, 21.5–62.1). *VHL* gene mutation was confirmed in seven patients. The patients’ demographic data, *VHL* gene mutations, and clinical VHLD history are provided in Table 1.

**Table 1.** Patient Characteristics and Clinical VHLD History

Patient	Sex	Age (Y)	*VHL* Gene Mutation	Further VHLD-Associated Tumors/Cysts	Prior RHs in Both Eyes, *n*
1	F	40.0	Unknown	None	5
2	F	49.6	Missense c.292T>C	PPGL	11
3	M	26.5	Deletion Exon 1	CNSH	0
				ccRCC	
				RC	
				PC	
4	M	31.5	Missense c.292T>C	CNSH	1
				PPGL	
				RC	
				PC	
				LC	
5	F	38.6	unknown	CNSH	9
				PNEN	
				LC	
6	F	57.8	Splice c.463+2 T>G	ccRCC	8
				PNEN	
				PC	
7	F	52.0	Unknown	ccRcc	OD: 8
				PNENRC	OS: Unknown due to enucleation 30 years ago
				LC	
8	M	21.5	Missense c.292T>C	None	OD: Unknown due to an old, still-existing retinal detachment OS: 3
9	F	62.1	Missense c.486C>G	CNSH	Unknown
				ccRCC	
				RC	
				PC	
10	F	35.6	Missense c.292T>C	PPGL	9

ccRCC, clear cell renal cell carcinoma; CNSH, central nerve system hemangioblastoma; F, female; LC, liver cyst; M, male; OD, right eye; OS, left eye; PC, pancreatic cyst; PPGL, pheochromocytoma/paraganglioma; PNEN, pancreatic neuroendocrine neoplasm; RC, renal cyst.

### Characteristics of Retinal Hemangioblastomas

**Figure 3.**
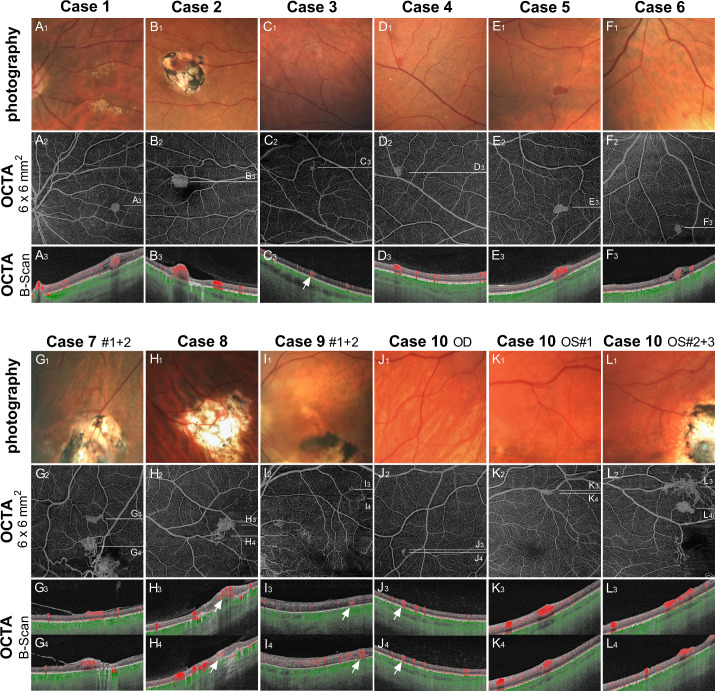
Overview of the retinal hemangioblastomas before treatment. Photography, en face OCTA 6 × 6 mm^2^ scans, and OCTA B-scans are shown for every case.

In total, 15 RHs were detected in 18 eyes: median one RH (range, zero to three) per eye and median one RH (range, one to four) per patient. None of the patients had a juxtapapillary RH or a peripherally located RH that could not be imaged by OCTA. All identified RHs are illustrated in Figure 3. Twelve RHs were primary, treatment-naïve tumors, and three were recurrent RHs located in pretreated areas. Of the 12 primary RHs, three were classified as exophytic, three as sessile, and six as endophytic. Five RHs had a tumor size of <0.25 DD, five between 0.25 and 0.5 DD, and five >0.5 DD. The morphology and size of each RH are described in Table 2.

**Table 2.** Laser Properties and Outcomes

Patient	Eye	Lesions, *n*	Morphology	Tumor Size (Disc Diameter)	Delivered Spots	Power Average (mW)	Total Energy (J)	Follow-Up (mo)	Regression (Complete, C; Partial, P; Unchanged, U)
1	OD	1	Ex	0.25–0.5	18	400	0.216	5.2	C
2	OD	1	Rec	>0.5	32	344.99	0.331	0.9	P
3	OD	1	S	<0.25	7	400	0.084	4.9	C
4	OD	1	En	<0.25	5	324	0.049	2.8	C
5	OS	1	S	0.25–0.5	18	400	0.216	5.3	C
6	OD	1	Ex	0.25–0.5	12	400	0.144	5.1	P
7	OD	1	En	0.25–0.5	10	400	0.12	2.6	P
7	OD	2	Rec	>0.5	18	400	0.216	2.6	C
8	OS	1	Rec	>0.5	31	323.23	0.301	3.3	U
9	OS	1	Ex	<0.25	2	400	0.024	2.5	C
9	OS	2	S	<0.25	5	400	0.06	2.5	C
10	OD	1	En	<0.25	4	400	0.048	3.2	C
10	OS	1	En	0.25–0.5	5	300	0.045	3.2	P
10	OS	2	En	>0.5	41	400	0.452	3.2	P
10	OS	3	En	>0.5	19	360	0.205	3.2	C

En, endophytic; Ex, exophytic; Rec, recurrent; S, sessile.

Proportions of complete regression, partial regression, and unchanged were calculated based on the number of lesions per eye.

### Laser Photocoagulation

A median of 12 laser spots (range, 2–41) of 215-µm diameter were delivered targeting the RH and feeding vessels. Treatment duration was 30 ms, and power per laser spot ranged between 240 and 400 mW. Median total applied energy per RH was 0.205 J (range, 0.024–0.452). Total applied energy per tumor correlated with tumor size (Spearman’s *r* = 0.79; 95% confidence interval [CI], 0.46–0.93; *P* < 0.001), with more energy applied to larger lesions. Detailed information on the laser treatment of each RH is provided in Table 2. Detailed laser treatment plans for each RH are depicted in Supplemental Figures S1.1 and S1.2.

### Evaluation of Treatment Success

**Figure 4.**
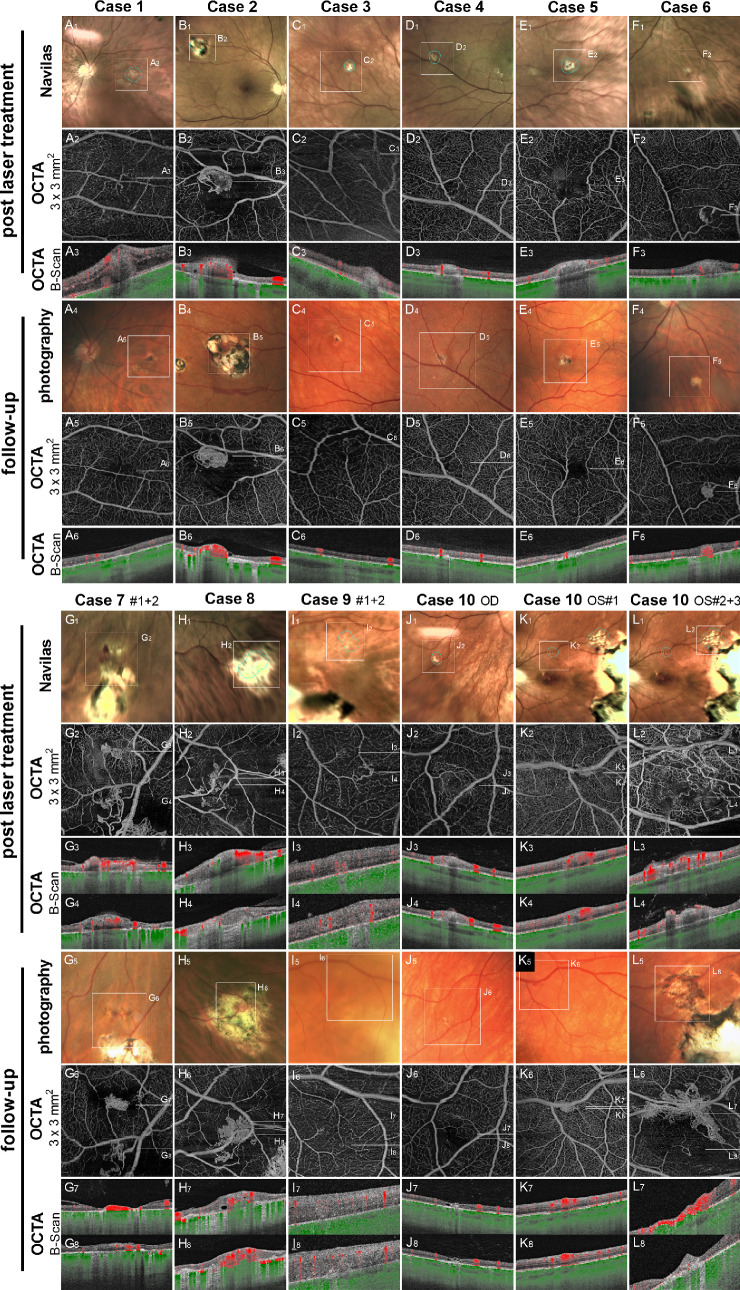
Overview of the retinal hemangioblastomas after treatment and follow-up. Photography, en face OCTA 3 × 3-mm^2^ scans, and OCTA B-scans are shown for every case after laser treatment (1–4) and at follow-up (5–8).

OCTA scans (3 × 3 mm^2^ and/or 6 × 6 mm^2^) of the treated areas performed directly after treatment revealed signs of residual tumor activity in four out of 15 lesions (cases 1, 2, 6, and 7#1). Three of these RHs (cases 1, 2, and 6) were retreated in the same session. Case 7#1 was not immediately retreated due to a small retinal hemorrhage during the laser session. Detailed imaging of treatment success is shown in Figure 4.

### Follow-Up

The first follow-up examination took place at a mean of 3.6 ± 1.5 months (range, 0.9–5.3) after laser treatment. Nine out of the 15 treated RHs (60%) exhibited complete regression, with no residual signal on OCTA and scarring on fundoscopy (Fig. 4, cases 1, 3, 4, and 5; Fig. 4, cases 7#2, 9#1+2, 10 OD, and 10 OS#3). Partial regression with a reduced but persistent OCTA signal was observed in five RHs (33.3%) (Fig. 4, cases 2 and 6; Fig. 4, cases 8 and 10 OS#1+2). One RH (6.7%) of case 7#1, which exhibited retinal hemorrhage during the laser session, remained unchanged at follow-up. Detailed outcomes for each lesion are provided in Table 2.

Four of the six RHs with persisting signs of activity (cases 2, 6, 8, and 10 OS#2) were retreated with laser photocoagulation at follow-up (OCTA-guided: case 2; conventional laser technique: cases 6, 8, and 10 OS#2), leading to complete regression at subsequent follow-ups. In case 10 OS#1, regression at follow-up and the high risk of vascular occlusion due to the proximity of the lesion to a large vein led to a decision for watchful observation and no further treatment. Follow-up of case 7#1 revealed a new RH close to the initial treatment area, and cryotherapy, including the area of the initial tumor, was successfully performed, resulting in a complete inactivity of the RHs.

Considering the time until complete inactivity of the RHs or, in the case of patient 10 OS#1, until the last check-up, the mean follow-up period was 10.3 ± 9.7 months (range, 2.8–33.8). In summary, laser treatment alone inactivated 13 out of 15 RHs (86.7%) with a mean of 1.3 ± 0.5 laser sessions per RH (range, one to two).

### Parameters Impacting Treatment Success After the First Laser Treatment

Complete regression was detected in one of three recurrent RHs (33.3%). Regarding the 12 primary RHs, complete regression occurred in two out of three exophytic RHs (66.6%), all three sessile RHs (100%), and three out of six endophytic RHs (50%), revealing no significant correlation for tumor morphology and treatment success (*P* = 0.32).

Complete regression occurred in all five RHs (100%) <0.25 DD in size, two of five RHs (40%) 0.25 to 0.5 DD in size, and two of five RHs (40%) >0.5 DD in size, demonstrating a trend of correlation of tumor size and treatment success (*P* = 0.08).

## Discussion

Widefield OCTA has been identified as a valuable supplementary tool for screening VHLD patients, revealing RHs that may not be discernible via ophthalmoscopy.^11^ However, our study introduces a novel technique that uses OCTA not only for screening but also for enhanced OCTA-guided navigated laser photocoagulation of RHs utilizing the Navilas Laser System. This approach enabled the treatment of lesions detected via OCTA, even those with limited visibility on ophthalmoscopy, thereby enhancing the efficacy and precision of therapeutic interventions.

### FA-, OCT-, and OCTA-Guided Laser Photocoagulation in Preexisting Literature

Several reports have described FA-, OCT-, and OCTA-guided laser photocoagulation using the Navilas for various retinal diseases. Amoroso et al.^23^ described a case series of nine eyes with advanced macular neovascularization treated with OCTA-guided laser therapy. The same group described a FA and/or OCT-guided micropulse laser treatment of 39 eyes with central serous chorioretinopathy.^24^ Similarly, Shin et al.^25^ described OCT-guided selective focal laser photocoagulation in 47 eyes with diabetic macular edema. These studies demonstrate OCTA/FA-guided laser photocoagulation as an effective and safe therapeutic approach, reducing the risk of excessive additional retinal damage compared to conventional laser photocoagulation.

### Parameters Influencing Treatment Success of OCTA-Guided Navigated Laser Photocoagulation of RHs Using the Navilas

Several factors may influence the success of laser treatment: (1) morphology and location of the RH, (2) tumor size, and (3) applied laser energy.

#### Morphology and Location of the Retinal Hemangioblastoma

Smid et al.^26^ described OCTA as an imaging tool for juxtapapillary RH classification in a single patient. However, data from a systematic application of OCTA imaging to differentiate RHs flow patterns in a larger series of patients are not yet available. In our study, we differentiated RH growth patterns using clinical appearance and OCTA B-scans. Because endophytic RHs are located superficially on the retina compared to exophytic RHs, which are embedded in deeper tissue, there is a conjecture that laser photocoagulation might exhibit greater efficacy for endophytic and sessile RHs. However, such a correlation could not be found in our study (*P* = 0.32).

#### Tumor Size and Applied Laser Energy

A significant correlation between tumor size and total laser energy was found in our study (*P* < 0.0001). Thus, only one of these two factors—tumor size—should be considered an influencing factor for the success of laser treatment. Although statistical significance was not attained due to a limited number of cases, a trend suggests that OCTA-guided laser photocoagulation is particularly effective for small RHs (*P* = 0.08). Notably, all five RHs with a tumor size of <0.25 DD exhibited complete remission after laser photocoagulation. These small RHs, potentially imperceptible or easily missed on ophthalmoscopy, have often been managed with a watchful waiting approach so far. However, our study demonstrates the precise and highly effective treatment of these small RHs using OCTA-guided laser photocoagulation, with minimal laser application (as low as two spots for case 9#1). This approach minimizes the risk of complications associated with laser treatment while addressing the tumor in its early stages.

### Reasons for Treatment Failure

Krivosic et al.^16^ described a success rate of 77% after a single laser session of 304 RHs in patients with VHLD and 97% using, if necessary, multiple laser treatments with a mean of 1.6 sessions per RH. In our study, only nine out of 15 RHs (60%) showed complete regression at first follow-up, and laser treatment alone was successful in 13 out of 15 RHs (86.7%) with a mean of 1.3 laser sessions per RH. In addition to the lower number of treated RHs in our study compared to Krivosic et al.,^16^ another explanation of the lower success rate after a single laser treatment might be that intraretinal fluid leads to shadowing artifacts of deeper retinal tissue in OCTA imaging.^27^^–^^29^ The edema induced by laser treatment in our study might have contributed to shadowing artifacts in the immediate OCTA assessment post-treatment, especially for larger tumors necessitating higher laser energy and thus resulting in larger edema. Another explanation may be that the laser-induced edema indirectly obstructed tumor vessels by applying pressure without permanently occluding them by scarring. Subsequent regression of edema could then restore blood flow in these tumors. Consequently, for larger RHs treated with OCTA-guided laser photocoagulation, we recommend a short-term follow-up examination when the post-laser edema has resolved to detect any residual activity that has been masked before.

### Limitations of the Study

One limitation of the study was the relatively low number of subjects and number of RHs. Growth over time of individual lesions was unknown, so later studies should investigate activity patterns, as these could show different reactions to laser treatments. Another limiting factor was that only one laser system was used, and no comparisons were made with other lasers or methods. Because OCTA can only visualize blood flow above a certain threshold, it is theoretically possible that a residual flow is present without this being able to be visualized in every case. It is also possible that the laser-induced edema of the retina may have reduced the OCTA imaging ability to be visualized. Systematic investigations would have to follow, but these are beyond the current feasibility study. Finally, it must be mentioned, that OCTA imaging of the peripheral retina is still lacking.^11^ Therefore, the described new technique of OCTA-guided navigated laser photocoagulation can only be used for RHs in the central or mid-peripheral retina.

## Conclusions

OCTA-guided laser photocoagulation of RHs in VHLD patients using enhanced Navilas is feasible. The proposed system showed to be precise and a safe therapeutic approach that allowed treatment of RHs in early stages minimizing potential complications caused by the laser treatment or the tumor. To further characterize and refine this emerging method, additional research with more patients is required. Such studies could investigate the optimal laser intensity required for specific tumor morphologies and sizes and may include randomized controls treated with a conventional laser.

## Supplementary Material

Supplement 1
