# Supplementary material for: Optical Coherence Tomography Angiography–Navigated Laser Photocoagulation of Retinal Hemangioblastomas in Patients With von Hippel–Lindau Disease
Source: Transl Vis Sci Technol. 2024 Jul 9;13(7):8. doi: 10.1167/tvst.13.7.8 (PMC11235141; doi:10.1167/tvst.13.7.8)
Supplement: Supplement 1 [file tvst-13-7-8_s001.pdf]

SUPPLEMENTAL FIGURES

Supplemental Figure 1.1 and 1.2: Planning the laser treatment for every retinal hemangioblastoma. Photography with NAVILAS® (1) En-face OCTA 6 x 6 mm^2 scan (2), NAVILAS®/OCTA overlay (3), NAVILAS®/OCTA overlay with planned laser spots (4), G NAVILAS® laser plan in treatment mode (5) for every retinal hemangioblastoma (A-L).

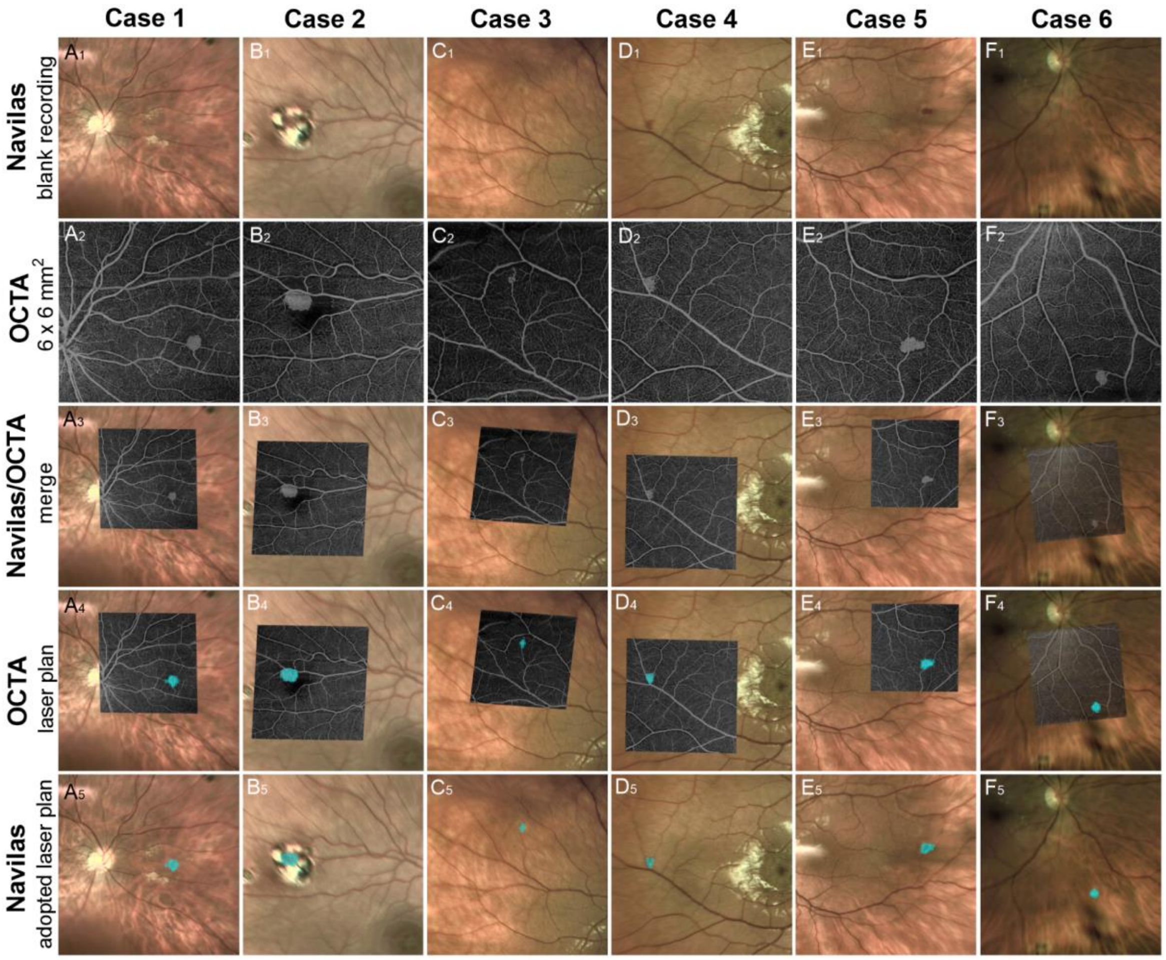

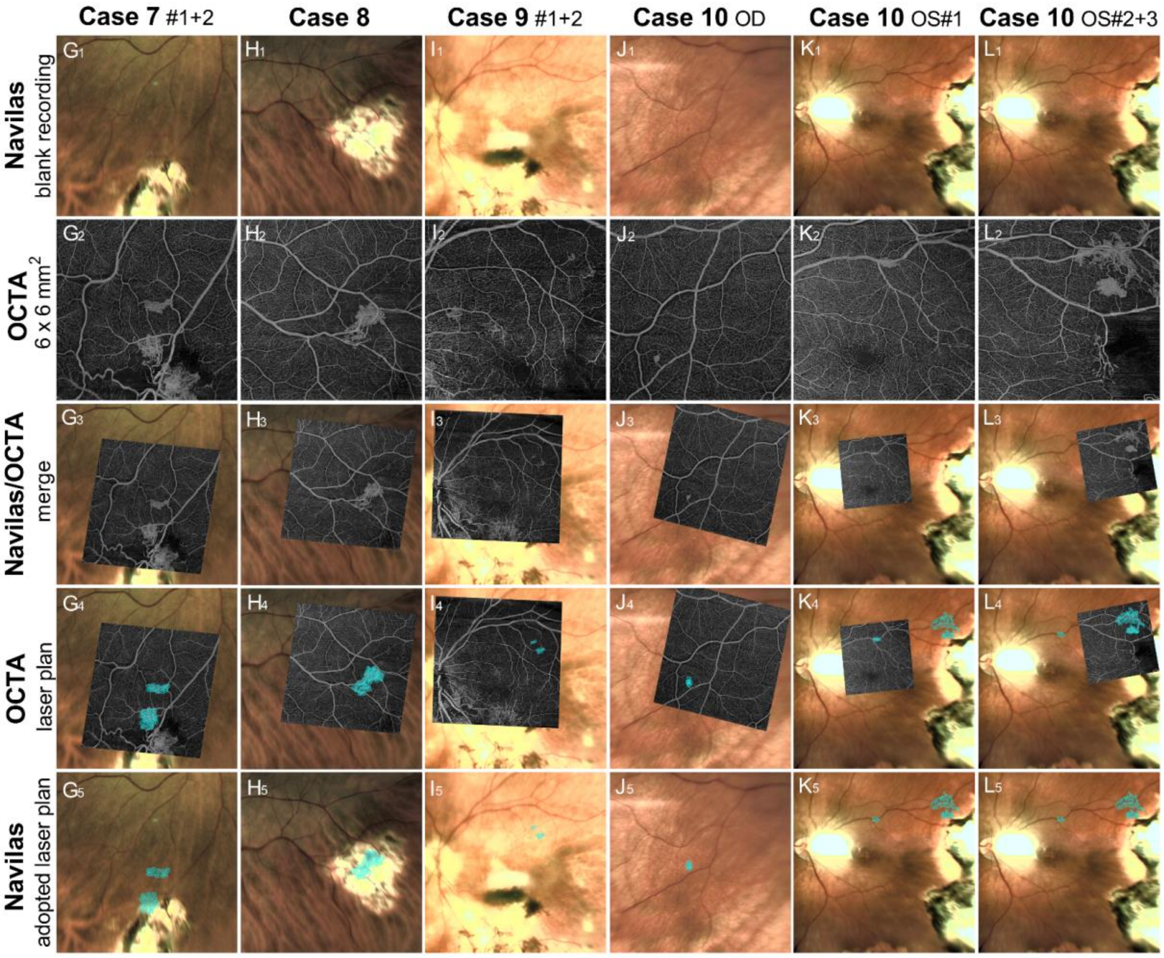
Case 7 #1+2
Case 8
Case 9 #1+2
Case 10 OD
Case 10 OS#1
Case 10 OS#2+3
Navilas
blank recording
OCTA
6 x 6 mm2
Navilas/OCTA
merge
OCTA
laser plan
Navilas
adopted laser plan
G1
H1
I1
J1
K1
L1
G2
H2
I2
J2
K2
L2
G3
H3
I3
J3
K3
L3
G4
H4
I4
J4
K4
L4
G5
H5
I5
J5
K5
L5
